# A 4D CT digital phantom of an individual human brain for perfusion analysis

**DOI:** 10.7717/peerj.2683

**Published:** 2016-11-30

**Authors:** Rashindra Manniesing, Christoph Brune, Bram van Ginneken, Mathias Prokop

**Affiliations:** 1 Department of Radiology and Nuclear Medicine, Radboud UMC, Nijmegen, The Netherlands; 2 Department of Applied Mathematics, University of Twente, Enschede, The Netherlands

**Keywords:** 4D CT, Digital phantom, Perfusion analysis, Acute stroke

## Abstract

Brain perfusion is of key importance to assess brain function. Modern CT scanners can acquire perfusion maps of the cerebral parenchyma *in vivo* at submillimeter resolution. These perfusion maps give insights into the hemodynamics of the cerebral parenchyma and are critical for example for treatment decisions in acute stroke. However, the relations between acquisition parameters, tissue attenuation curves, and perfusion values are still poorly understood and cannot be unraveled by studies involving humans because of ethical concerns. We present a 4D CT digital phantom specific for an individual human brain to analyze these relations in a bottom-up fashion. Validation of the signal and noise components was based on 1,000 phantom simulations of 20 patient imaging data. This framework was applied to quantitatively assess the relation between radiation dose and perfusion values, and to quantify the signal-to-noise ratios of penumbra regions with decreasing sizes in white and gray matter. This is the first 4D CT digital phantom that enables to address clinical questions without having to expose the patient to additional radiation dose.

## Introduction

Only as of recently, Computed Tomography (CT) scanners are capable of four-dimensional (4D) and *in vivo* imaging of the complete human brain at submillimeter resolution. Visualizing the contrast dynamics at this level of detail makes it possible to quantify and study the hemodynamics of the cerebral parenchyma. This is of key importance to assess brain function; for example, in the case of acute stroke. In ischemia, a large perfusion defect is an important contra-indication for thrombolytic treatment.

The connection between contrast dynamics and hemodynamic parameters such as cerebral blood flow (CBF), cerebral blood volume (CBV) and mean transit time (MTT) has been established by the indicator dilution theory (Zierler, 1962). Central in that theory is the notion of an impulse function *h*(*t*) describing the probability distribution of transit times through the system. The exact shape of this function is unknown, but several imposed parametrizations have led to clinically relevant perfusion maps and increased understanding of neurovascular pathology (Bivard et al., 2011; Murphy et al., 2008; Parsons et al., 2007). It has sparked an ongoing interest in the search of optimal thresholds to define the regions with perfusion defects (Bivard et al., 2013; Rekik et al., 2012); in particular, the tissue at risk (penumbra), potentially salvageable by thrombolytic treatment, and the tissue already irreversibly damaged (infarct core).

Even though the indicator dilution theory has widely been accepted, the algorithm steps to get from 4D imaging data to perfusion maps are complex and many factors have been reported contributing to variability in perfusion values not reflecting true hemodynamic variations. These factors include the temporal sampling rate of the acquisition protocols (Wintermark et al., 2004), the selection of the arterial input and venous output functions (Sanelli et al., 2004; Soares et al., 2009), the type and implementation of the software that is used for perfusion calculations (Sanelli et al., 2007; Kudo et al., 2010; Fahmi et al., 2012), and the total radiation dose utilized in the CT acquisition protocols (Manniesing et al., 2016). Not all factors have been identified nor have their influences on perfusion values and clinical interpretation been rigorously assessed.

Variability higher than 27% was reported on identical patient imaging data (Soares et al., 2009), and significant differences were found among commercially available software for perfusion calculations (Kudo et al., 2010). Clearly, without a proper and quantitative understanding of the relations between these factors and perfusion values, the search for optimal thresholds is futile. This is the main reason CT perfusion imaging still raises controversies, and why it has not been widely adopted as a standard diagnostic modality for stroke management in clinical practice (Liebeskind et al., 2015).

This problem has been recognized by the Stroke Imaging Research (STIR) group. In 2013, STIR published a roadmap on acute stroke imaging research for the next five years (Wintermark et al., 2013). One of their recommendation is to use a digital phantom of the human brain to validate and objectively compare perfusion analysis methods. Their assumption is that such a model provides an objective reference standard of hemodynamic values similarly as a real physical phantom does for e.g., scanner calibration of Hounsfield Units (HU) and for radiation dosimetry.

**Figure 1:**
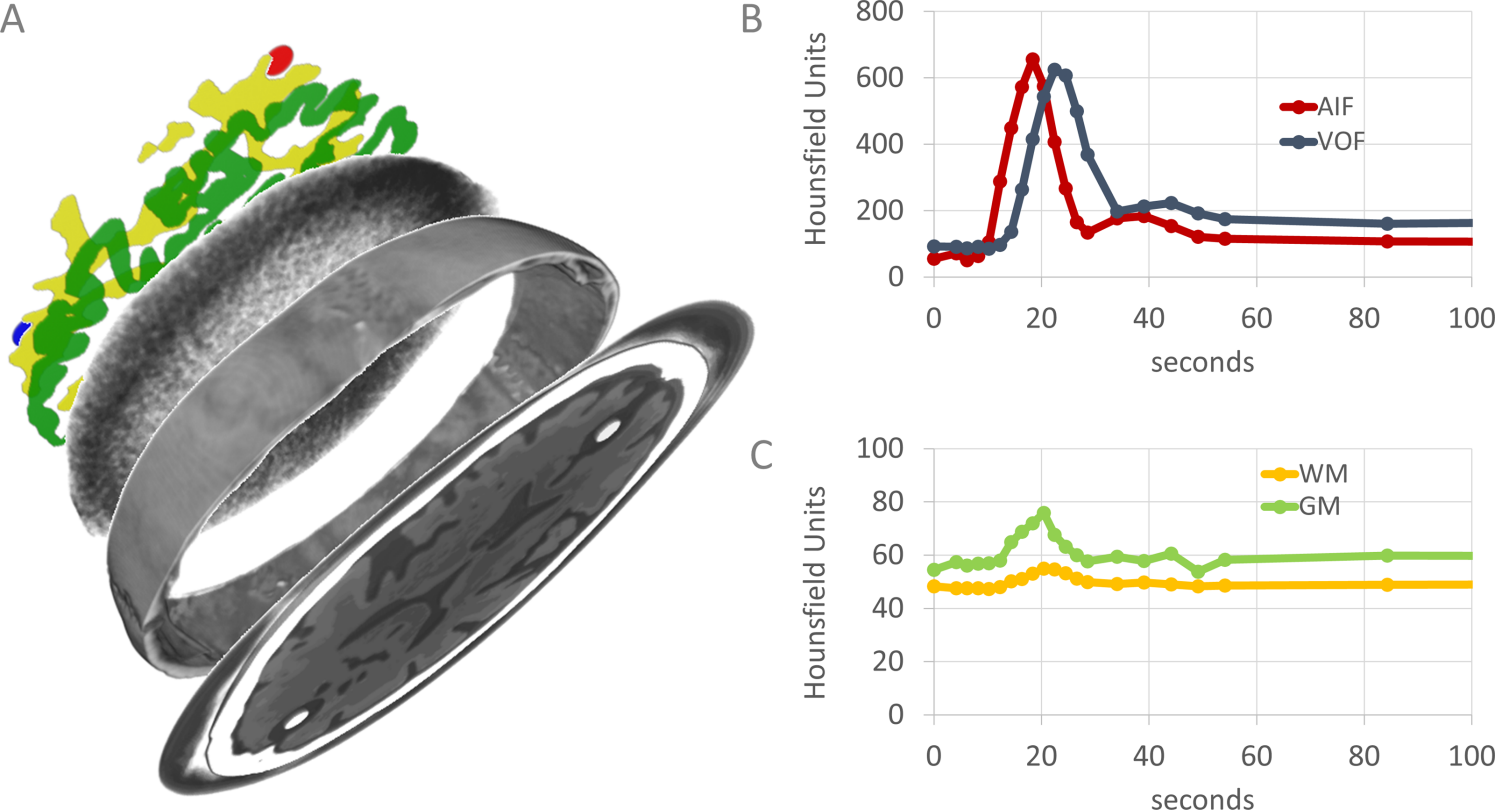
Graphical overview of the 4D CT digital phantom. Rendering (A) shows the phantom decomposed into its main components. From top to bottom: artery and vein (red and blue), WM (yellow), GM (green), part of the noise pattern, part of the outer skull. Each component can be modified and then are merged to represent a new 4D digital representation of the brain (lowest slice, only one axial slice is shown). The spatial content of the phantom is defined in 3D, but some components are only visualized in 2D. Graphs (B) and (C) show typical examples of contrast dynamics (visualized separately, but intrinsic part of the phantom). The AIF (red) and VOF (blue) usually have higher intensity values than the brain parenchyma. The dots represent the time points of the volumetric CT acquisitions.

In this work we present a 4D CT digital phantom of the human brain. It is a comprehensive framework that first makes a full decomposition of 4D patient information into signal, noise and morphology. Subsequently, each component can be modified and then are merged to create a new 4D digital representation of the brain (Fig. 1). In this manner, any type of acquisition protocol and patients with any type and degree of stroke related pathology can be simulated to quantitatively and qualitatively study the effect on perfusion values.

In literature three related but limited digital phantoms have been proposed to date (Riordan et al., 2011; Pianykh, 2012; Kudo et al., 2013). None have the full specifications and flexibility as proposed in this work (Table 1). In particular, previous phantoms rely on simulated tissue attenuation curves only to represent contrast dynamics of the parenchyma and vasculature, whereas our model has the option to use patient-specific attenuation curves. This way patient group variability can be studied (retrospectively) and, for example, questions like how perfusion values change if the patient would have been scanned with a different acquisition protocol can be answered without having to expose the patient to additional radiation dose. Our phantom therefore supersedes all existing phantoms to date and is the first to address clinical questions.

**Table 1:** Overview of related work.

Model	Patient	Signal			Noise		Morphology	
	Specific	Measured	Simulated	Penumbra	Measured	Simulated	Anatomical	Geometrical
Riordan et al. (2011)			•	•	•		•	
Pianykh (2012)			•			•		•
Kudo et al. (2013)			•			•		•
This work	•	•	•	•	•	•	•	•

The goal of this paper is to present and validate the new 4D CT digital phantom. In addition we present two clinical applications.

## Methods and Materials

The proposed 4D CT digital phantom consists of three main components: signal, noise and morphology. The perfusion maps were calculated with a publicly available and vendor independent software package.

### Signal

#### Measured

A patient specific digital phantom is constructed by adding tissue attenuation curves from a 4D CT image from that patient. CT imaging was performed on a 320-detector row CT scanner (Toshiba Aquilion ONE; Toshiba Medical Systems Corporation, Japan). The acquisition protocol started 5 s after contrast injection with a volumetric scan at 200 mAs exposure, followed after 4 s by 14 scans one every 2 s at 100 mAs, followed by 5 scans, one every 5 s at 75 mAs. Each volumetric scan had 16 cm coverage and was made at 80 kV at 0.5 s rotation time. Each volumetric image had 512 × 512 × 320 voxels with 0.5 × 0.5 × 0.5 mm voxel size.

The tissue attenuation curves are sampled by manually annotating regions of interest with high confidence that it belongs to a certain tissue type. Boundary voxels and thus partial volume voxels are excluded. In this work, annotations were carried out by one radiologist in training with approximately 5 years of experience with 4D CT images. Normal appearing WM and normal appearing GM in the basal ganglia were annotated on 5 mm slabs of the temporal average image. The AIF was annotated in the M1 segment of the middle cerebral artery at the unaffected side and the VOF was annotated in the sinus sagittalis superior, both on 5 mm slabs of the temporal maximum intensity projection image. In order to simulate a CT acquisition protocol with arbitrary temporal sampling rate, a linear interpolation or gamma variate (Thompson et al., 1964) can be selected.

#### Simulated

Tissue attenuation curves are simulated following Ostergaard et al. (1996b):

(1) \begin{eqnarray*}C(t)=\mathrm{CBF}\cdot R(t)\otimes \mathrm{AIF}(t).\end{eqnarray*}

With *C*(*t*) the tissue attenuation curve, CBF the cerebral blood flow, *R*(*t*) the residue function describing the amount of contrast still present in a volume at time *t*, and AIF(*t*) the arterial input function simulated using a gamma variate function:

(2) \begin{eqnarray*}\mathrm{AIF}(t)= \left\{ \begin{array}{@{}ll@{}} \displaystyle 0\hspace*{10.00002pt}&\displaystyle t\leq {t}_{0}\\ \displaystyle {C}_{0}(t-{t}_{0})^{a}{e}^{-(t-{t}_{0})/b}\hspace*{10.00002pt}&\displaystyle t\gt {t}_{0}. \end{array} \right. \end{eqnarray*}

With *C*_0_ = 1.0, *a* = 3.0, *b* = 1.5 s and *t*_0_ = 12 s. The VOF was simulated with the same gamma variate function with *t*_0_ = 16 s. The residue function was defined as exponentional function:

(3) \begin{eqnarray*}R(t)={e}^{- \frac{t}{MTT} }.\end{eqnarray*}

With MTT the mean transit time. Given a CBF and MTT, the CBV then follows from the central volume principle:

(4) \begin{eqnarray*}\mathrm{CBF}= \frac{\mathrm{CBV }}{\mathrm{MTT}} .\end{eqnarray*}

Thus, given user-defined values for any subset two of CBF, CBV and MTT, a tissue attenuation curve *C*(*t*) can be simulated.

#### Penumbra

Penumbra tissue is simulated by smoothing the attenuation curves of healthy tissue such that the peak intensity value is reduced while maintaining the area under the curve. This approach is based on the central volume principle (Eq. (4)) and the approximation of blood volume by the area under the curve Hoeffner et al. (2004). Penumbra will then have decreased CBF, normal CBV and increased MTT. In this work we reduced the peak intensity value by 50% of the measured curves to simulate penumbra.

### Noise

#### Measured

There are three sources of noise in CT imaging: quantum noise, electronic noise and structural noise. Quantum noise is caused by the Poisson distributed fluctuations of the photons in the X-ray beam, electronic noise is caused by the electronic read out of the detector elements, and structural noise is caused by the spatially fixed variation of the gain leading to e.g., ring artifacts. In normal clinical routine, quantum noise is the dominating factor and is inversely proportional to the square root of the exposure. The exposure is one of the main targets for minimization when designing and optimizing CT acquisition protocols. The noise of a CT scanner is measured by scanning a 3D anthropomorphic head phantom consisting of an epoxy filled human skull. The epoxy has approximately the same density as brain tissue. In this work, the head phantom was scanned at in total 23 exposure settings in the range [10, 300] mAs, with each setting repeated 31 times to obtain the random fluctuation of noise, with remaining scan settings similar to scan settings of the patients. Between each scan, the CT tube was let to cool down to standard operating temperature. A new CT acquisition protocol of a 4D digital brain model is constructed by taking one of the 31 scans for each volumetric time point for a given exposure setting; in this way, multiple permutations of the same CT acquisition protocol can be constructed.

To solely add the noise and not the density of the epoxy itself to a new 4D digital phantom, the mean intensity value of epoxy was shifted to zero HU. The mean intensity was obtained by averaging the 31 scans of the head phantom at the highest exposure (hence lowest noise) at 230 mAs, within the epoxy area defined by an interval threshold of −200 and 600 HU. The mean intensity was then subtracted from this epoxy area in every scan at every exposure setting. Full details of this step are described in Van den Boom et al. (2014).

#### Simulated

**Figure 2:**
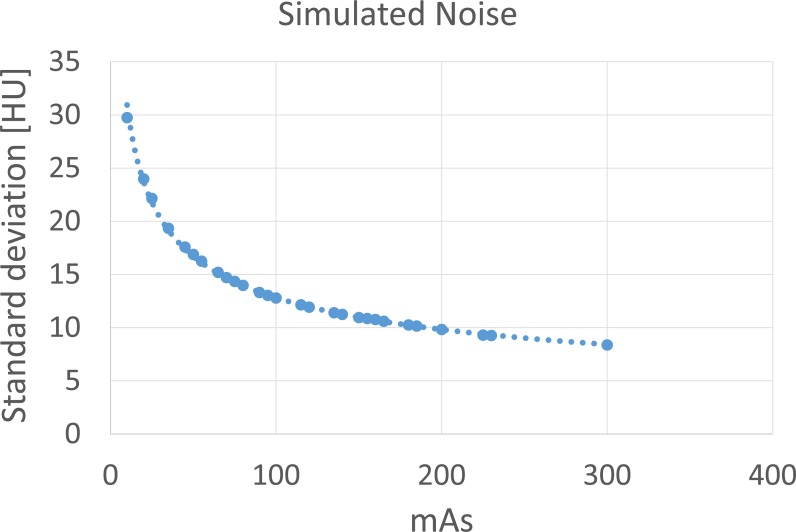
Standard deviation of a small region of interest at the center location of the anthropomorphic head phantom scanned a different exposures. An exponential function is fitted through the data points.

Simulated noise is based on a Gaussian distribution with its standard deviation depending on the desired exposure setting. The relation between exposure and standard deviation was found by an exponential fit through the available pairs of data points with the standard deviation taken of a small region of interest at the center location of each scan of the head phantom at different exposures (Fig. 2). These scans were only used to collect data points of the standard deviation versus exposure and were not used otherwise in the constructed phantom, in case of simulated noise.

### Morphology

The digital phantom has several options to define the 3D spatial content. Most commonly used perfusion analysis software packages at least expect a region representing the AIF, the VOF, one tissue attenuation curve and the presence of surrounding skull tissue. Often those software packages search at fixed spatial locations relative to the skull to automatically find the AIF and VOF, or they use the skull to spatially align the 4D data over time. The skull was obtained by volumetric CT acquisition of the anthropomorphic head phantom resulting in 512 × 512 × 320 matrix with voxel size of 0.5 × 0.5 × 0.5 mm, which was resampled to 32 slices of 5.0 mm. Two cylindrical objects were then placed: one anterior to simulate an artery and one posterior to simulate a vein.

The intra-cavity space of the skull was then filled with parenchymal tissue. Several options for the parenchyma are available: a realistic 3D anatomical representation obtained from a pre-segmented and publicly available magnetic resonance (MR) atlas of the brain (Rohlfing et al., 2010). This atlas was constructed from T1 weighted MR images of 24 normal subjects which were segmented into WM, GM and CSF and registered to a common space. The MR atlas was rigidly registered to the intra-cavity space of the skull. The second option is to select a geometrical morphology consisting of maximum of nine cylindrical objects equally spaced within the skull and with user-defined diameters that can be set independently. The final option is to select homogenous tissue type or two-tissue type separated at the midsagittal plane to represent the whole brain or the right and left hemisphere, respectively. The latter is important to study the uniformity or symmetry of perfusion maps calculated by a software package.

For all boundary voxels adjacent to different tissue types, partial volume effects were simulated by spatial smoothing with a Gaussian kernel with standard deviation of 1.5 mm.

### Perfusion analysis

Perfusion maps were calculated with the publicly available and vendor independent software package Perfusion Mismatch Analyzer (PMA), version 5.0.0.0, developed by the Acute Stroke Imaging Standardization Group (ASIST), Japan (http://asist.umin.jp/index-e.htm). We used the delay-insensitive block-circulant singular value decomposition (bSVD) (Wu et al., 2003), with the following options: 256 × 256 matrix, 5 mm slabs, smoothing turned on, and arterial input function rescaling on with the venous output function. All other settings were kept at default values.

The spatial content that was defined geometrically or by the MR brain atlas and that was used to construct the digital phantom is also used to measure the perfusion values per tissue type in the corresponding perfusion maps.

## Experiments and Results

The digital phantom contains several options and depending on the application at hand a specific configuration can be selected. An overview of the configurations used in this work is given in Table 2. Five studies are presented of which two have been published separately (Van den Boom et al., 2014; Manniesing et al., 2016).

**Table 2:** Overview of the different phantom configurations of the five studies (A–E) presented in this work. Signal can be measured (M), simulated (S) or in case of perfusion defect, simulated from a measured signal (SM); noise can be measured (M) or simulated (S), and morphology can be anatomical (A) or geometrical (G).

Study		#Patients	#Phantoms	WM/GM	AIF/VOF	Noise	Morphology
A Validation	Van den Boom et al. (2014)	20	200	M	M	M	A
B Validation		20	400	M	M	S	A
C Validation		20	400	S	S	M	A
D Application	Manniesing et al. (2016)	20	2,000	M	M	M	A
E Application		10	200	SM	M	M	G

### (A) Validation: measured signal and noise versus patient data

The first validation study addresses how well the digital phantom constructed from an individual patient represents the cerebral perfusion values of that patient. To this end, we have quantitatively compared signal and noise values of both white matter (WM) and gray matter (GM) of 20 ischemic stroke patients to their corresponding phantoms. For each patient, 10 random permutations of the acquisition protocol were simulated to capture the random variation of noise. Thus, in total 200 phantoms were built. Intraclass correlation coefficients were in the range of 0.92 and 0.99 for the mean perfusion values (signal) and in the range of 0.86 and 0.93 for the standard deviations (noise). Linear fits showed slope values between 0.90 and 1.06. We concluded that a realistic patient specific digital phantom based on measured signal and measured noise is feasible.

### (B) Validation: measured noise versus simulated noise

**Figure 3:**
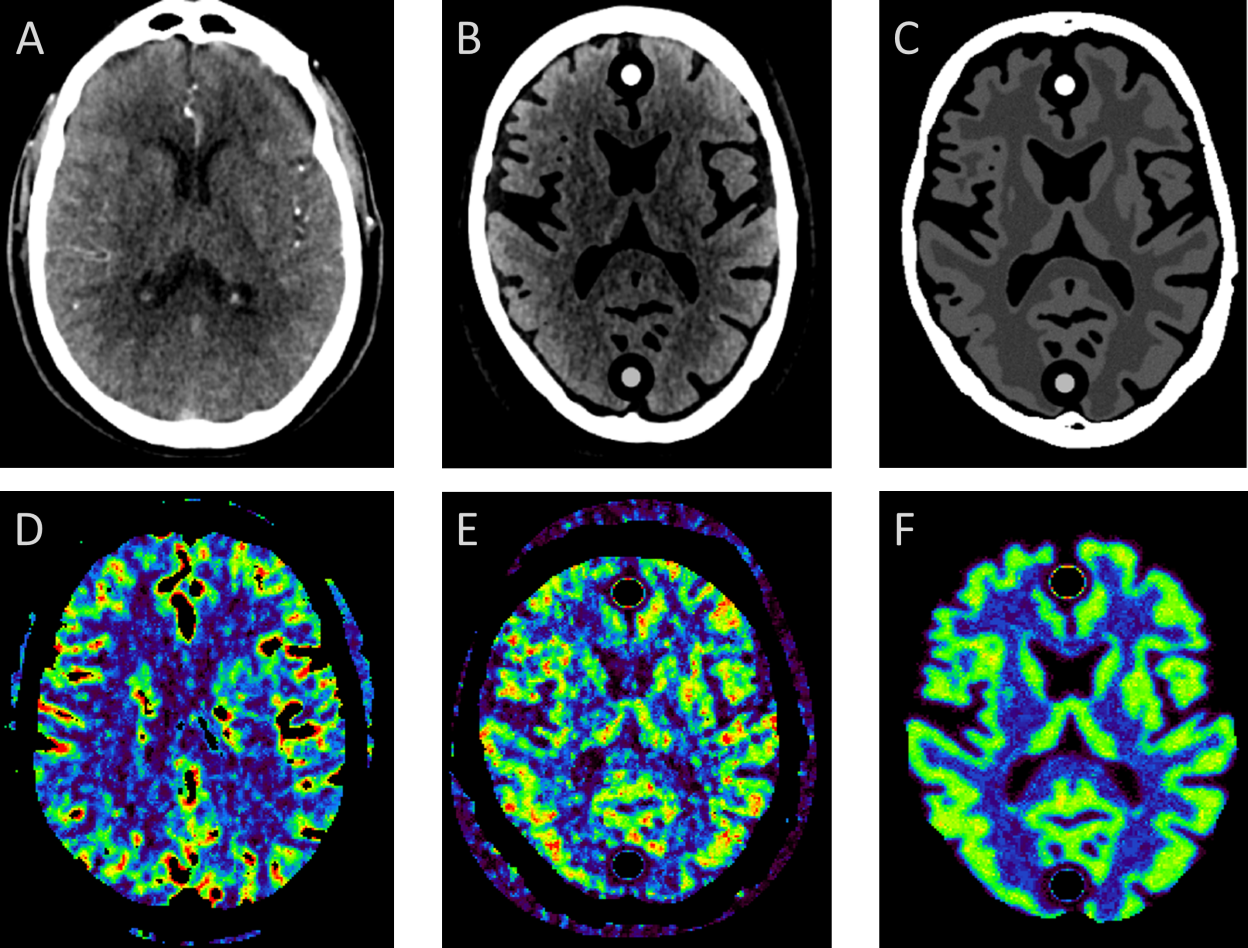
Comparing measured with simulated noise. (A) and (D) show the original patient data and corresponding CBF map; (B) and (E) show the corresponding digital phantom with measured noise and measured tissue attenuation curves; (C) and (F) the same phantom but with simulated Gaussian distributed noise. Cross sectional slices at approximately the level of the basal ganglia are shown of the 4D CT patient and 4D digital phantom data.

Noise can approximated by adding a Gaussian noise distribution in the image domain. This is the approach adopted by all related work on digital phantoms, except one (Riordan et al., 2011), and in many (clinical) applications (Uwano et al., 2011; Sasaki et al., 2012; Fang, Chen & Sanelli, 2012; Kudo et al., 2013; Fang, Chen & Sanelli, 2013). In this study, we set to compare both approaches, by taking the patient specific digital phantom as defined in (A) and changing the noise patterns by simulated noise using a Gaussian distribution (Fig. 3).

In this study 20 consecutive patients (seven men, 13 women, mean age 62 years, age range 35–88 years) with a clinical diagnosis of acute stroke were included. Institutional review board approval was waived for the retrospective use of patient imaging data in this work.

For each patient, and for each noise type, 10 random permutations of acquisition protocol were simulated to capture the random variation of noise. Thus in total 400 phantoms were built. For each patient, the average perfusion map of the 10 permutations were taken as the representative perfusion map. We then compared CBF values of the original patient data, which served as reference standard, with the CBF values in WM and GM of both sets. In the comparison, we took the mean ± standard deviation CBF in the patient, in the regions manually annotated by the observer, which was already done in the first step to measure the tissue attenuation curves, and the mean ± standard deviation CBF of the digital phantom in the region defined by the pre-segmented anatomical masks. Thus, we compare signal (mean) and noise (standard deviation) of both approaches. A slope value closer to one is considered closer to the reference standard.

**Figure 4:**
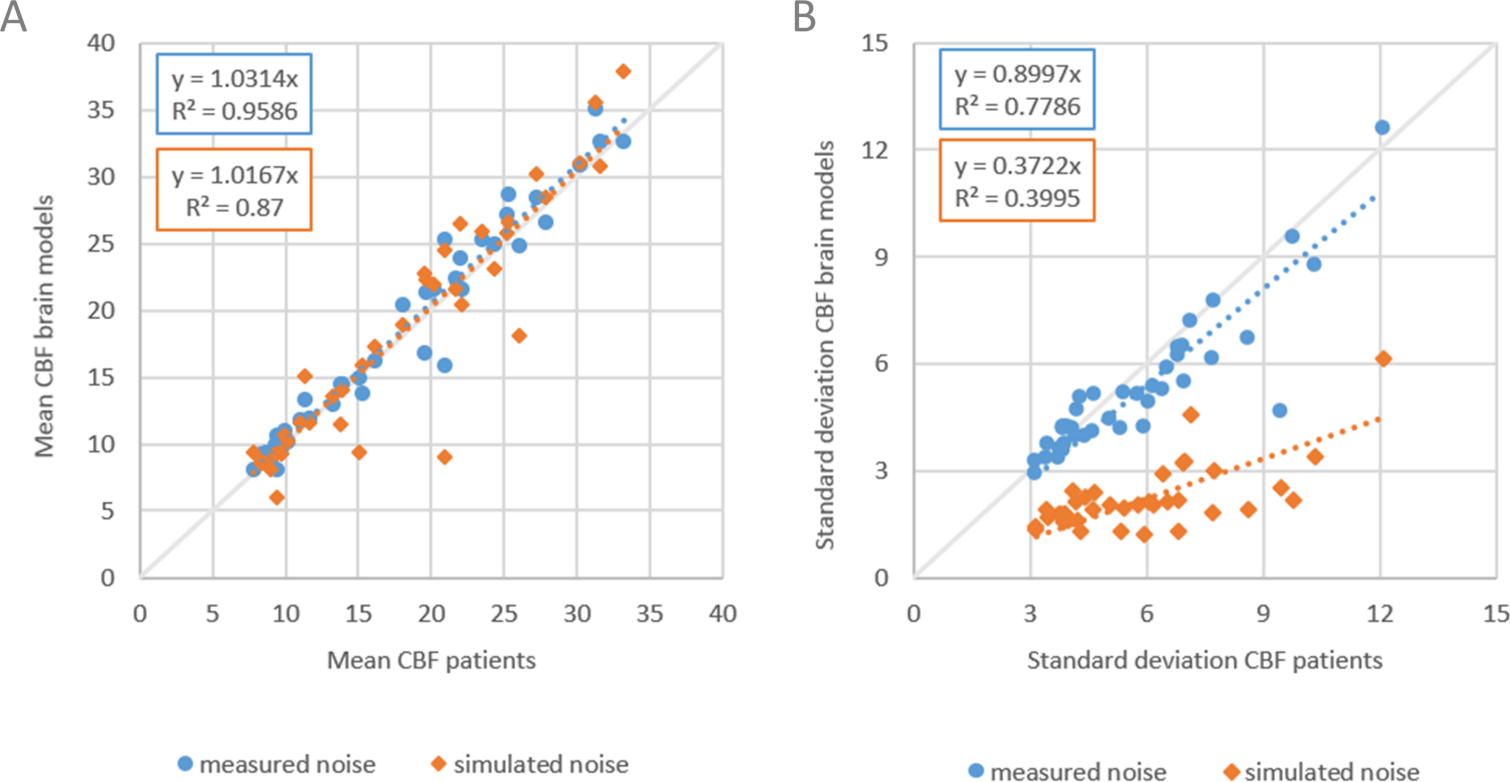
Comparing measured with simulated noise. The mean and standard deviation CBF of WM and GM of patient data versus corresponding digital phantoms with measured noise and simulated noise. (A) Dashed lines indicate linear fits with slope values of 1.03 and 1.02 for measured and simulated noise respectively, (B) slope values were 0.90 and 0.37 respectively.

The results are shown in Fig. 4. Slope values of the linear fits for the means were similar with 1.03 and 1.02 for measured and simulated noise, respectively. Slope values for the standard deviations were 0.90 and 0.37, respectively. The mean CBF values are similar (Fig. 4A) but the standard deviations show large differences for measured versus simulated noise (Fig. 4B).

Thus, Gaussian noise in the CT image propagates less realistically to the final perfusion maps. We recommend to use digital phantoms with measured noise. Measuring noise by scanning an anthropomorphic head phantom captures many important properties of the CT acquisition and image reconstruction, including the cupping artifacts in head CT as a result of beam hardening, which are very difficult to simulate realistically, otherwise.

### (C) Validation: measured signal versus simulated signal

**Figure 5:**
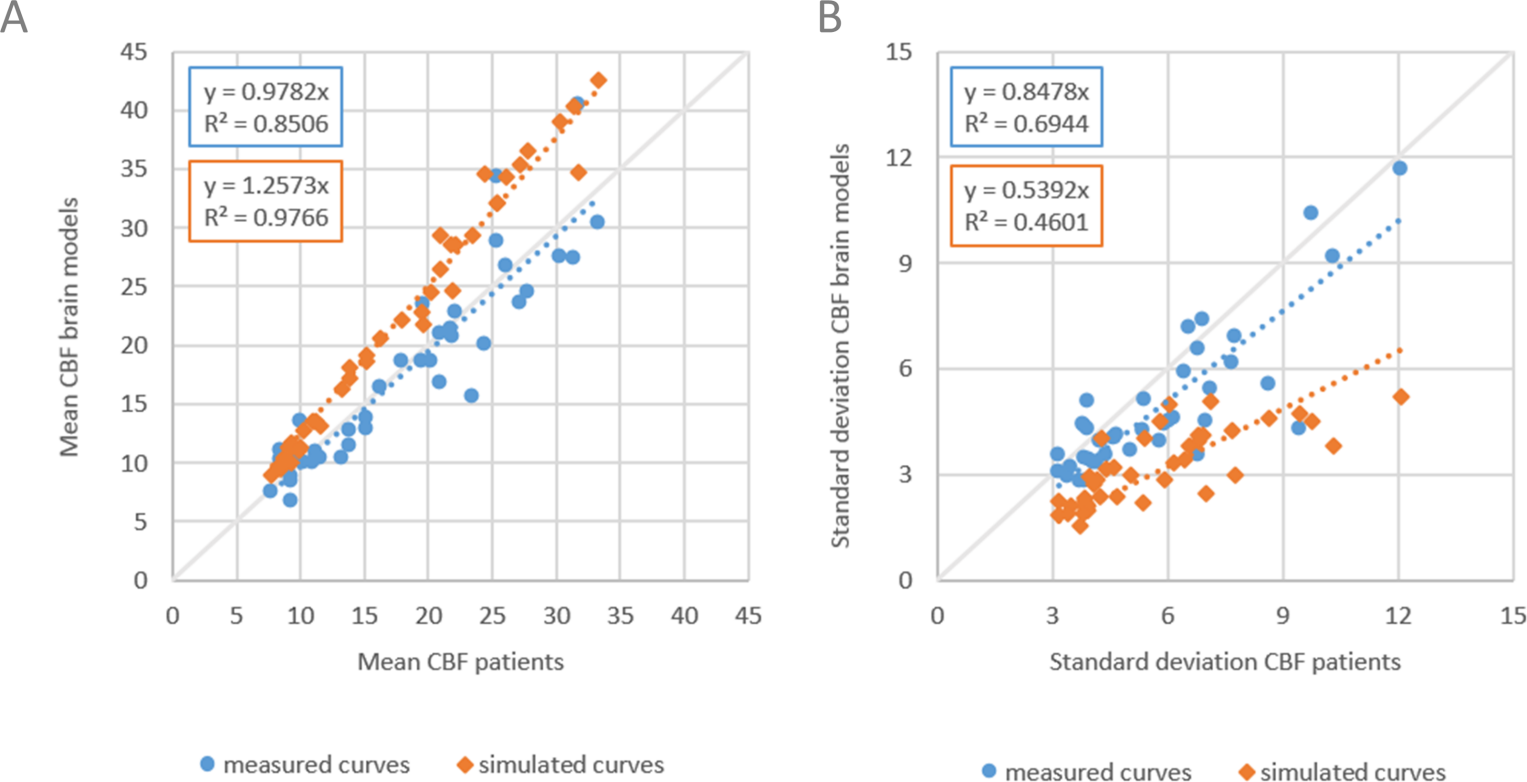
Comparing measured with simulated curves. The mean and standard deviation CBF of WM and GM of patient data versus corresponding digital phantoms with measured tissue attenuation curves and simulated curves based on the indicator dilution theory. (A) Dashed lines indicate linear fits with slope values of 0.98 and 1.26 for measured and simulated curves respectively, (B) slope values were 0.85 and 0.54 respectively.

In a subsequent study we set to compare digital phantoms with measured and simulated signal. The simulated signal is based on applying the indicator dilution theory to generate tissue attenuation curves, instead of solving it to find perfusion values, and is the common approach followed in related work to build digital phantoms (Table 1). To minimize other sources of variation in the comparison, we started with measured signal from the patient data that were used in set A, and the calculated perfusion values. The resulting CBV and MTT were used as input to generate tissue attenuation curves, with other parameter settings equal to what was used by Kudo et al. (2013). We followed the same steps as in the previous study: inclusion of the same 20 consecutive ischemic stroke patients, construction of two sets of phantoms, one as in (A), and one with simulated curves (set C), 10 random permutations of the acquisition protocol to capture random variation of noise, bSVD method for perfusion calculations, and same quantitative comparisons of mean and standard deviations of CBF, with the perfusion maps from the original patient data serving as reference standard. Again, another set of in total 400 digital phantoms were built. The results are shown in Fig. 5. Slope values of the linear fits for the means were 0.98 and 1.26 for measured and simulated curves, respectively, and for the standard deviations 0.85 and 0.54 respectively. Values closer to one are considered closer to the reference standard.

Thus, digital phantoms with measured tissue attenuation curves leads to more realistic CBF perfusion values than phantoms with simulated curves. The discrepancy is caused by the arbitrary choice of parameter values and parametrization of the residue function in the indicator dilution. Different settings will lead to different tissue attenuation curves given the same input perfusion values. The settings that coincide with the settings used in the perfusion analysis software, most likely will produce perfusion values closer to the perfusion values that were used to generate the curves.

### (D) Application: dose dependency of perfusion values

Radiation is harmful for the patient. CT perfusion imaging is associated with high radiation dose because multiple volumetric acquisitions are required. It is important to reach as low as reasonably achievable exposure to ensure patient safety, however, until recently it was unknown how radiation dose, and hence image noise, would influence the calculated perfusion values. In the following application, we have quantitatively assessed the relation between radiation dose and perfusion values by a stepwise reduction of the total radiation dose of a CTP protocol. Twenty consecutive patients with a clinical diagnosis of acute stroke received a CTP with an average effective dose of 5 mSv. Digital phantoms were constructed specifically for each patient to simulate the same acquisition protocol at a lower total radiation dose in the range [0.5, 5.0] mSv with step size of 0.5 mSv. The phantoms were constructed with measured noise and measured tissue attenuation curves. CBF, CBV and MTT values were calculated for each patient at each dose setting. With 10 permutations per digital phantom, the total number of phantoms used in this study was 2,000. The optimum total radiation dose was defined as the minimum dose for which the maximum mean difference of all perfusion values remained within 5% of the reference standard at 5.0 mSv.

We found that perfusion values CBF, CBV and MTT increase for decreasing dose and that below 2.0 mSv there is marked increase in particular for WM. The maximum mean differences for WM and GM between 5.0 mSv and 2.5 mSv were 4.5% for CBF, 5.0% for CBV and 1.9% for MTT. At 2.0 mSv these were 15.6%, 18.1% and 3.4%, respectively. We concluded that the total radiation dose can be lowered to 2.5 mSv with only minor quantitative effects on cerebral perfusion values: an important result for the stroke patient and radiological practice.

### (E) Application: penumbra quantification

Perfusion loss due to blood flow disturbance in case of ischemia leads to tissue damage (oligemia and penumbra) and irreversible damage (infarct core) if the flow is not restored in time. Treatment is targeted at restoring the blood flow and preventing penumbra from turning into infarct core by intra-venous thrombolysis or endovascular thrombectomy. Penumbra is therefore an important biomedical imaging marker. Intensive research is carried out on the validation and the sensitivity of detecting penumbra in CT perfusion imaging. In this application the digital phantom is used to quantitatively assess the contrast-to-noise ratios (CNR) of penumbra of decreasing size at standard clinical radiation dose. Ten consecutive patients with a clinical diagnosis of acute ischemia were scanned with the same CT acquisition protocol as described in “(B) Validation: measured noise versus simulated noise.” Digital phantoms were constructed specifically for each patient and with 10 permutations of the acquisition protocol to capture the random variation of noise. The foreground of the digital phantom contained cylindrical objects with decreasing diameters (from 30 mm to 1 mm, total objects 18) representing penumbra and the background consisted of healthy WM tissue curves. Similar phantoms were constructed for GM. Thus in total 200 digital phantoms were used in this study. Perfusion values were calculated with PMA ASIST.

**Figure 6:**
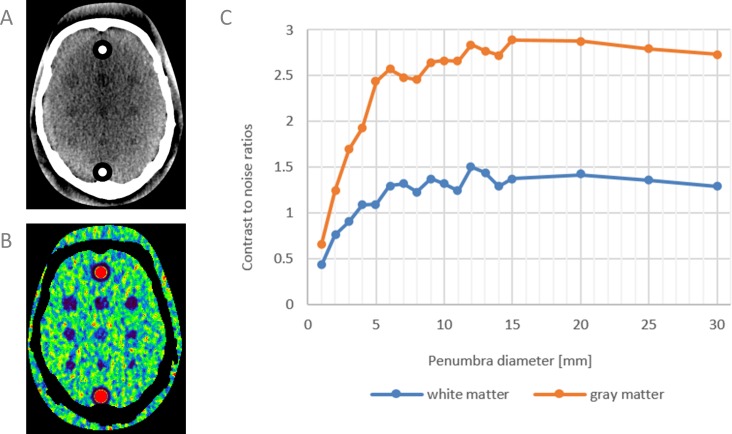
Contrast-to-noise ratios of simulated penumbra. (A) and (B) simulated perfusion loss (penumbra) with decreasing diameters on healthy white matter, and corresponding CBF map. One cross sectional slice of the 4D digital phantom is shown. Similar phantoms were constructed for GM (not shown). (C) CNRs of penumbra versus background of WM and GM as function of penumbra diameter.

The results showed that for this acquisition protocol and this software package, a peak intensity reduction of 50% of the healthy tissue curves led to an average CBF reduction of 68% for WM and 82% for GM (CBF values were averaged over all penumbra diameters). CNRs are plotted in Fig. 6. At similar object sizes, the CNR for WM is approximately half the CNR of GM. This is the first study in which the sensitivity of CT perfusion imaging has been quantitatively assessed for penumbra visualization.

## Discussion

A flexible framework for constructing realistic 4D CT digital phantoms has been presented and validated to study parenchymal perfusion. It has a wide range of technical and clinical applications. The framework is a direct answer to the request by the international Stroke Imaging Research (STIR) group to develop a digital phantom in order to validate and objectively compare perfusion analysis methods. However, we go beyond their envisioned purpose as the range of application is wider than validations and comparisons alone. The following is a non-exhaustive list ordered from technical to clinical applications.

The first group of applications concerns the optimization of the CT acquisition protocol. CT uses ionizing X-rays for imaging which is harmful for the patient. The challenge is to strike the right balance between acceptable levels of radiation dose and CNR. This tradeoff cannot be explored on patients. With the digital phantom questions can be addressed as how radiation dose should be distributed over the number of 3D acquisition time points, how many acquisition time points are required at a certain amount of dose, or what is the optimal temporal sampling rate.

The second group of applications concerns the study of the properties (e.g., robustness, stability) of perfusion algorithms and the development of new perfusion algorithms. There is no consensus in the choice of algorithms nor in their parameters when deriving perfusion values from time attenuation curves. Several deconvolution methods and numerical schemes have been proposed, including standard singular value decomposition (sSVD) (Ostergaard et al., 1996b; Ostergaard et al., 1996a), bSVD (Wu et al., 2003), reformulated SVD (rSVD) (Smith et al., 2004) and a latest addition, the Bayesian approach (Mouridsen et al., 2006). All algorithms estimate or parametrize the residue function *R*(*t*), which is equal to one minus the integral of the unknown impulse function *h*(*t*) describing the fraction of contrast still remaining in the system. Different methods leads to different perfusion maps with identical input data (Sanelli et al., 2007; Kudo et al., 2010; Fahmi et al., 2012), a disturbing finding for clinical practice. There are no definite answers which method should be used, but the digital phantom provides a direct mean to investigate these properties. For example, by duplicating the dose dependency study (Manniesing et al., 2016) for different perfusion analysis methods, the difference of their responses can be quantified and their robustness to noise can be assessed.

The third group of applications concerns the study of the hemodynamics of the cerebral parenchyma. Because the digital phantom is first fully decomposed, each aspect can be modified and the relation to perfusion values can be assessed. In other words, all factors from acquisition and reconstruction, to image processing and clinical interpretation, potentially contributing to variability of perfusion values can quantitatively be assessed. A critical aspect is the shape of the tissue attenuation curves of the arteries and the parenchyma. The curves are subject to noise and often multiple curves in a small region of interest are averaged to minimize the noise. But this leads to curve dispersion (Calamante, Gadian & Connelly, 2000). Another approach is model fitting, which has the additional advantage of obtained interpolated values between time points, which is a prerequisite for the current perfusion analysis methods when dealing with non-equidistantly time sampling intervals. The current standard is the gamma-variate model (Thompson et al., 1964). This model was proposed because it visually resembles a measured tissue attenuation curve. The gamma-variate however, ignores the recirculation effect and assumes constant and the same intensity values before and after the peak. With the digital phantom, time curve fits beyond the gamma-variate can be explored. Deviations from normal time curves are important in diagnostic imaging. The digital phantom allows to study the influence of reduced perfusion (or infarcted region) for a wide range of pathological related parameters. What exactly constitutes a perfusion defect and what is the minimum size still observable given a maximum total radiation dose? In this work we have shown how controlled reduced time curves lead to penumbra tissue and we have shown for the first time the relation between penumbra size and CNRs for WM and GM.

The framework provides an option to add simulated noise following a Gaussian distribution. Even though more realistic simulations can be obtained by considering more advanced methods, e.g., Joemai, Geleijns & Veldkamp, 2010; Žabić et al., 2013, Gaussian noise is used in many (clinical) studies (Uwano et al., 2011; Sasaki et al., 2012; Fang, Chen & Sanelli, 2012; Kudo et al., 2013; Fang, Chen & Sanelli, 2013) and is therefore included in our work. The framework also provides an option to add simulated tissue attenuation curves by application of the indicator dilution theory, and is the approach adopted by all related work. This approach suggests a reference standard of true perfusion values, which is not the case (Colton & Kress, 1983; Kaipio & Somersalo, 2006), and may therefore lead to invalid conclusions. Despite these limitations, both options were supported in order to carry out validation studies.

Validation of the digital phantom is important. We have compared the signal and noise characteristics with patient data in three separate studies, and concluded that the use of measured noise by scanning an anthropomorphic head phantom and measured tissue attenuation curves by manual annotating regions of interest, leads to more realistic phantoms than simulated noise and signal. This is an important finding as all proposed digital phantoms in literature use simulated noise and simulated curves, except only one work which uses measured noise (but simulated curves) (Riordan et al., 2011).

The drawback of using measured noise and curves is practicality. To obtain noise characteristics of a CT scanner, multiple acquisitions of a physical head phantom and subsequent image processing are required, and to obtain measures tissue attenuation curves, manual annotations are required. Despite this, to ensure that studies based on digital brain models have clinical value and sound implications, we advocate the use of measured noise and measured curves in all studies involving patient data. There is an additional argument as well: measuring the noise enables to study the scanner specific contribution to variability of perfusion values.

This work has one main limitation which is fundamental of nature and applies to all digital phantoms with simulated curves using the indicator dilution theory (of note, the proposed framework supports both simulated and measures curves in order to compare our digital phantom with existing phantoms). A pitfall observed in related work is the assumption that the digital phantom represents true perfusion values. This is not the case. The digital phantom provides an input reference standard, and therefore an objective comparison of the responses can be made, but no decisive conclusions can be drawn whether one of them is closer to the true values than the other.

The reason is that it does not directly represent a real perfusion value (e.g., CBV, CBF or MTT), but it does so indirectly, inferred from the underlying residue function and parameters of the indicator dilution theory which translate to a tissue curve. In fact, one can easily show that given a set of perfusion values and selection of residue function, a family of tissue curves can be generated (just use other values of the unknowns, e.g., the hematocrit variables). But also the other way around, a tissue curve fed into the indicator dilution theory can produce a set of perfusion values. Which one is then the true value? It is important to realize that even if consensus is reached in adopting the indicator dilution theory as the best way of modelling hemodynamics and in choosing some reasonable values of the unknowns of this theory, e.g., the shape of the residue function, there is still a fundamental problem at the heart of this, which is called an inverse crime (Colton & Kress, 1983; Kaipio & Somersalo, 2006).

The inverse crime denotes the act of employing the same model to generate data (from perfusion values to tissue curves), as well as to invert data (from tissue curves to perfusion values). This should be avoided to prevent trivial inversions and therefore unrealistically optimistic results. Thus, validating and comparing perfusion analysis methods using any digital phantom with simulated curves should be done with the greatest care. This type of phantom does not provide an objective reference standard or true perfusion values, unlike a real physical phantom does for other purposes.

Generally, to address clinical questions using the digital phantom, the following steps should be taken: First, if measured tissue curves are used, defining the inclusion and exclusion criteria of patients and manual annotating regions of interest. Second, if measured noise is used, scanning the anthropomorphic head phantom at pre-defined exposure settings (depending on which acquisition protocol needs to be simulated) to extract the scanner noise characteristics, and for each exposure doing this multiple times to capture the random variation of noise. Finally, selecting the desired method for perfusion calculation. The exact configuration of the digital phantom depends on the clinical question that needs be addressed. Example configurations are given in this work (Table 2).

Future work includes expanding the options to define spatial content of the phantom, by developing algorithms to segment the anatomical structures in 4D CT including WM, GM, CSF, arteries, veins and skull. These segmentations enables a more realistic representation and can be used to sample the tissue attenuation curves at various locations in the brain.

In conclusion, the proposed framework to construct 4D digital digital phantoms is flexible and versatile, and allows to study the hemodynamics of the cerebral parenchyma in CT in a bottom-up fashion.

## Supplemental Information

10.7717/peerj.2683/supp-1 Supplemental Information 1 Results (tables and figures) from experiments described in paper

## Additional Information and Declarations

### Data Availability

The following information was supplied regarding data availability: The raw data has been supplied as Supplemental Dataset.
